# Sodium content in processed food items in Sweden compared to other countries: a cross-sectional multinational study

**DOI:** 10.3389/fpubh.2023.1182132

**Published:** 2023-06-08

**Authors:** Karin Karlsson, Karin Rådholm, Elizabeth Dunford, Jason H. Y. Wu, Bruce Neal, Johan Sundström

**Affiliations:** ^1^Department of Medical Sciences, Uppsala University, Uppsala, Sweden; ^2^The George Institute for Global Health, University of New South Wales, Sydney, NSW, Australia; ^3^Department of Health, Medicine and Caring Sciences, Linköping University, Linköping, Sweden; ^4^Department of Nutrition, Gillings Global School of Public Health, The University of North Carolina at Chapel Hill, Chapel Hill, NC, United States; ^5^The School of Population Health, University of New South Wales, Sydney, NSW, Australia

**Keywords:** sodium, salt, processed food, packaged food, FoodSwitch, nutrition, Sweden

## Abstract

**Background:**

Dietary sodium has a dose-response relationship with cardiovascular disease, and sodium intake in Sweden exceeds national and international recommendations. Two thirds of dietary sodium intake comes from processed foods, and adults in Sweden eat more processed foods than any other European country. We hypothesized that sodium content in processed foods is higher in Sweden than in other countries. The aim of this study was to investigate sodium content in processed food items in Sweden, and how it differs from Australia, France, Hong Kong, South Africa, the United Kingdom and the United States.

**Methods:**

Data were collected from retailers by trained research staff using standardized methods. Data were categorized into 10 food categories and compared using Kruskal-Wallis test of ranks. Sodium content in the food items was compared in mg sodium per 100 g of product, based on the nutritional content labels on the packages.

**Results:**

Compared to other countries, Sweden had among the highest sodium content in the “dairy” and “convenience foods” categories, but among the lowest in “cereal and grain products,” “seafood and seafood products” and “snack foods” categories. Australia had the overall lowest sodium content, and the US the overall highest. The highest sodium content in most analyzed countries was found in the “meat and meat products” category. The highest median sodium content in any category was found among “sauces, dips, spreads and dressings” in Hong Kong.

**Conclusion:**

The sodium content differed substantially between countries in all food categories, although contrary to our hypothesis, processed foods overall had lower sodium content in Sweden than in most other included countries. Sodium content in processed food was nonetheless high also in Sweden, and especially so in increasingly consumed food categories, such as “convenience foods”.

## 1. Introduction

An excessive intake of dietary sodium is linked to an increased risk of cardiovascular diseases (CVDs) (1–3), largely mediated by an increased blood pressure (2). In 2019, it was estimated that high sodium intake alone attributed to 1.89 million deaths globally (4). In Sweden, high sodium intake approximated accounted for 20% of all myocardial infarctions and 10% of all strokes (5). Reducing sodium intake has recently been convincingly demonstrated to reduce the risk of CVDs and mortality (6). The main contributors to dietary sodium intake in Sweden, as well as many other countries, are processed or ultra-processed food items (7–9). Previous studies from other countries estimate that processed foods generally account for 66–80% of population dietary sodium intake, with the remainder deriving from salt added at the table or during cooking (10–12). Packaged processed- and ultra-processed foods are often high in sodium, saturated fat and sugar (13–15).

The Nordic Nutritional Recommendations (NNR) and the Swedish Food Agency recommend a maximum intake of 6 g salt (2,400 mg sodium) per day (16, 17). The only current initiative to lower sodium intake is the “green keyhole,” a front-of-pack label for consumers that indicates the best in class products, which have lower sodium, saturated fat, sugar as well as higher fiber content. The criteria for putting the label on a food item differs between food categories (18). The Swedish Food Agency also provides information on their website on why it is important to lower sodium intake and suggestions on how to do it (19). The current estimated intake of dietary salt in Sweden is 10–12 g (4,160–5,000 mg sodium) per day per capita (20, 21), and adults in Sweden have been shown to be among the highest consumers of ultra-processed foods in Europe (22). Consumption of ultra-processed foods in Sweden has increased significantly in recent decades (23) simultaneously as consumption of freshly prepared meals has decreased (23, 24).

One way to lower population sodium intake is to develop food policies targeting food manufacturers and encouraging the lowering of sodium content in their products (25–27). To better understand if this would be an effective strategy, and how such types of policies should be formulated, the sodium content in commercially available food items needs to be reviewed. The aim of this study was to examine the sodium content in processed food items in Sweden and how it differs from that in other countries. No previous studies have examined the content of sodium in food items in Sweden compared to other countries on an extensive food category level. The hypothesis of this study was that processed food items in Sweden contain more sodium than comparable foods in other countries. The specific research questions were: (1) How much sodium do processed food items in Sweden contain? and (2) How does sodium content in processed food items in Sweden differ from that in other countries with FoodSwitch data (Australia, France, Hong Kong, South Africa, the United Kingdom and the United States), and how does sodium content differ in processed foods across food categories?

## 2. Materials and methods

### 2.1. Study design

This was an observational, cross-sectional and multinational study examining sodium content in Swedish processed food items, and comparing the sodium content in processed food items from Australia, France, Hong Kong, South Africa, the United Kingdom and the United States.

### 2.2. Data collection

The Swedish data were collected for this study in 2021. Data were extracted from four large Swedish retailer databases: DABAS, ICA, Coop and City Gross. The Swedish data were manually categorized by researchers with the Department of Medical Sciences at Uppsala University. Data were categorized according to the FoodSwitch categorization system to be comparable with the other datasets.

For this project, the available FoodSwitch data from six countries were included for comparison. Countries were selected to ensure a broad geographical comparison to the Swedish data. The George Institute for Global Health's FoodSwitch Branded Food Composition Database contains nutrition information for packaged foods from multiple countries. Product information in FoodSwitch is controlled with an established quality assurance protocol, reviewed and categorized into the database (28). Further details of collection and extraction of data for each specific country within the FoodSwitch database are as follows: Australia: Collected in 2013–2022, extracted in 2022; France: Collected in 2021, extracted in 2022; Hong Kong: Collected in 2017–2019, extracted in 2022; South Africa: Collected in 2016–2021, extracted in 2022; UK: Collected in 2013–2020, extracted in 2022.

### 2.3. Food categories

The selected food categories were influenced by the WHO benchmarks for sodium content in food, published in 2021 (29). Analyses were examined using 10 broad food categories: “bread and bakery products,” “cereal and grain products,” “convenience foods,” “dairy,” “edible oils and oil emulsions,” “fruits, vegetables, nuts and legumes,” “meat and meat products,” “sauces, dressings, dips and spreads,” “seafood and seafood products” and “snack foods.” A description of food items in the included categories can be found in Supplementary Table 1. Sodium content in the food items was compared in mg sodium per 100 g of product, based on the nutritional content labels on the packages.

### 2.4. Statistical analysis

Statistical analyses were conducted using the statistical software Stata (17.0, StataCorp. LLC, College Station, TX). Kruskal–Wallis test of ranks followed by the *post-hoc* test Dunn's pairwise comparisons of means were used to determine differences in distribution of sodium content between countries. The *post-hoc* test, Dunn's pairwise comparison of means test, where the rank sum means are compared to each other (30), was made with a Benjamini–Hochberg procedure to keep the false discovery rate at 5%.

Items were excluded if they had missing values for sodium content, if the value for sodium was zero or if the value was incorrect. The incorrect values were identified by comparing items, specifically extreme outliers, with the nutritional information on the manufacturer website. Additionally, the following food types, that mainly consists of sodium, were excluded: “herbs,” “herb pastes,” “curry powder mixes,” “herbs and spices,” “spices,” “seasonings,” “salt,” “baking soda,” “baking powder” and “bicarb soda.”

A country was defined as having sodium contents higher or lower than most countries if the sodium values were higher or lower than >5 countries.

### 2.5. Ethical considerations

This study used secondary food composition data, for which ethical approval is not needed. The data are not considered sensitive and cannot be linked to individuals.

## 3. Results

### 3.1. Derivation of the study database

**Figure 1:**
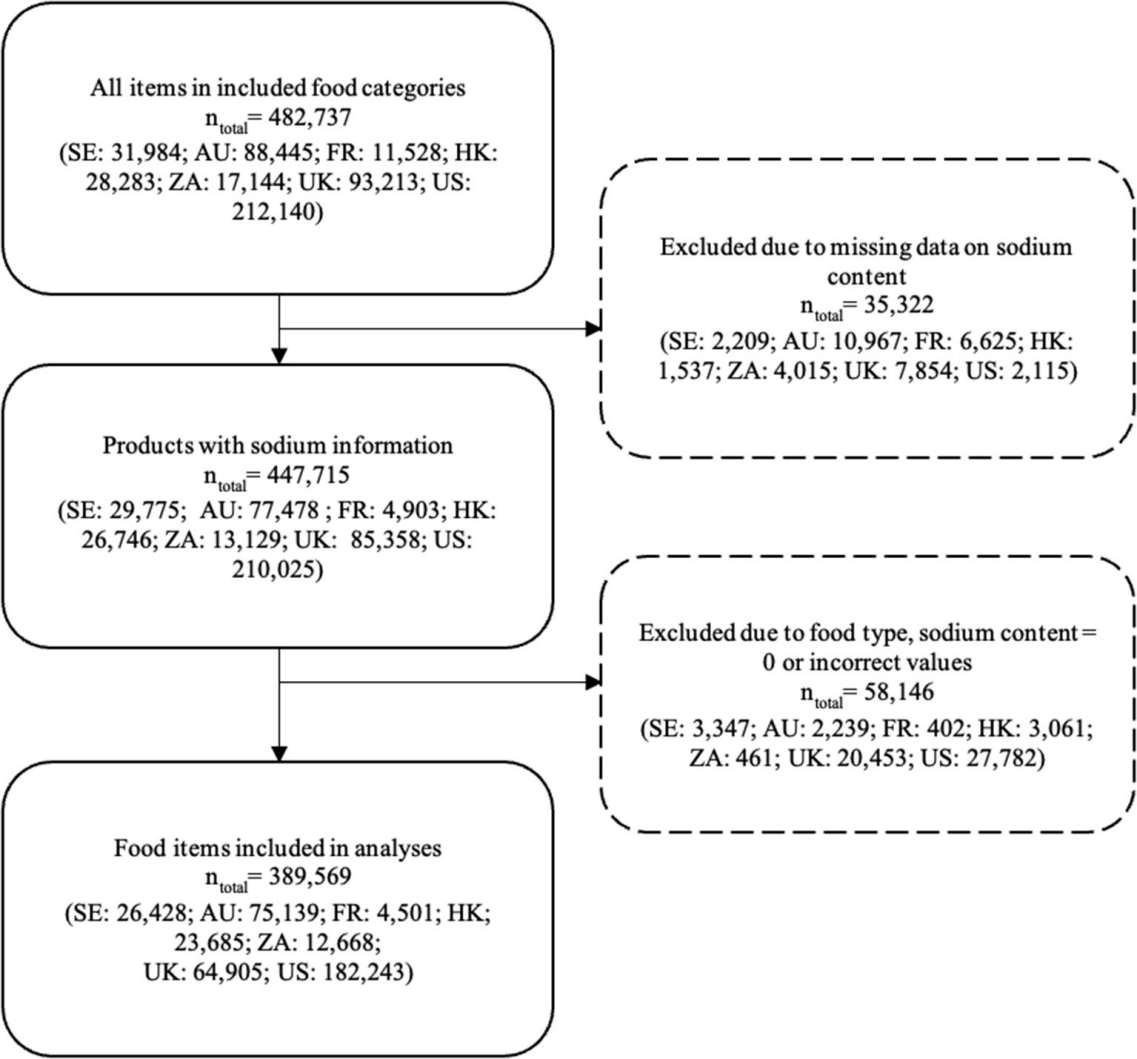
Flowchart illustrating the exclusion of data. SE, Sweden; AU, Australia; FR, France; HK, the Hong Kong Special Administrative Region of the People's Republic of China; ZA, South Africa; UK, United Kingdom; US, United States.

In total, the multinational database had 482,737 items in the included food categories. After removal of missing data, zero values, incorrect values and sub-categories the total study sample was *n* = 3,89,596 (see Figure 1).

### 3.2. Sodium content in processed foods by country

The sodium content in each food category is described by country in Table 1.

**Table 1:** Characteristics of sodium content in each food category by country.

	**Sweden**		**Australia**		**France**		**Hong Kong**		**South Africa**		**United Kingdom**		**United States**	
	***N***	**mg sodium/ 100 g, median (Q1; Q3)**	***N***	**mg sodium/ 100 g, median (Q1; Q3)**	***N***	**mg sodium/ 100 g, median (Q1; Q3)**	**N**	**mg sodium/ 100 g, median (Q1; Q3)**	***N***	**mg sodium/ 100 g, median (Q1; Q3)**	***N***	**mg sodium/ 100 g, median (Q1; Q3)**	***N***	**mg sodium/ 100 g, median (Q1; Q3)**
Bread and bakery products	3,859	380 (212; 509)	12,133	350 (220; 483)	394	420 (246; 594)	3,966	290 (159; 460)	1,631	382 (264; 560)	11,545	200 (90; 360)	30,053	385 (267; 529)
Cereal and grain products	1,347	40 (4; 280)	6,461	43 (7; 292)	342	25 (9; 339)	3,874	264 (20; 1,280)	1,907	110 (8; 323)	4,557	152 (40; 290)	12,680	357 (150; 613)
Convenience foods	1,148	424 (320; 546)	7,727	300 (230; 426)	810	318 (271; 424)	1,948	389 (230; 592)	1,036	434 (303; 939)	10,273	232 (180; 320)	16,092	376 (259; 551)
Dairy	4,607	124 (42; 600)	12,401	50 (41; 420)	1,134	59 (42; 424)	3,021	53 (39; 120)	1,891	50 (40; 288)	8,875	100 (50; 600)	29,463	103 (54; 607)
Edible oils and oil emulsions	254	509 (424; 600)	841	340 (5; 400)	86	170 (21; 848)	172	445 (36; 680)	321	360 (5; 450)	530	520 (360; 600)	777	643 (607; 750)
Fruits, vegetables, nuts and legumes	3,393	64 (20; 382)	10,317	26 (5; 270)	467	212 (21; 369)	2,600	132 (13; 459)	2,193	36 (9; 276)	5,384	120 (40; 323)	35,699	151 (18; 385)
Meat and meat products	6,314	721 (339; 975)	7,926	530 (348; 917)	520	763 (509; 1,102)	1,298	634 (536; 1,020)	1,241	634 (508; 947)	11,076	400 (240; 650)	15,836	732 (353; 1,054)
Sauces, dressings, dips and spreads	2,266	636 (382; 1,320)	9,071	536 (305; 1,080	348	551 (369; 1,102)	3,287	1,200 (430; 3,244)	1,411	655 (357; 1,800)	7,313	440 (300; 800)	23,532	600 (372; 968)
Seafood and seafood products	2,815	297 (81; 721)	3,108	400 (284; 558)	297	466 (424; 1,187)	1,013	520 (352; 810)	374	360 (266; 457)	2,014	400 (260; 600)	5,731	329 (152; 500)
Snack foods	425	520 (200; 720)	5,154	440 (193; 670)	103	585 (509; 746)	2,506	615 (433; 902)	663	634 (480; 839)	3,338	586 (440; 800)	12,380	607 (405; 893)

Q1, 1st quartile, 25 %; Q3, 3rd quartile, 75%; Greener colors are lower sodium values and redder colors are higher sodium values; Hong Kong, the Hong Kong Special Administrative Region of the People's Republic of China.

The processed food categories in Sweden had the following ranking from highest median sodium content to lowest: “meat and meat products,” “sauces, dressings, dips and spreads,” “snack foods,” “edible oils and oil emulsions,” “convenience foods,” “bread and bakery products,” “seafood and seafood products,” “dairy,” “fruits, vegetables, nuts and legumes” and “cereal and grain products.”

Table 2 shows the ranking of each country in relation to each other based on median sodium content in the different food categories, with the highest rank indicating the highest sodium content. Based on the rankings in Table 2, Australia had the overall lowest sodium content while the United States had the highest. The rankings also show that Sweden had among the lowest sodium content in the “seafood and seafood products,” “cereal and grain products” and “snack foods,” and overall ranked with the third lowest sodium content of all countries. The highest sodium contents in Sweden, compared to the other countries, were identified in the “dairy” and “convenience foods” categories.

**Table 2:** Ranking of countries in each category based on the median values of sodium content.

	**Sweden**	**Australia**	**France**	**Hong Kong**	**South Africa**	**United Kingdom**	**United States**
Bread and bakery products	4	3	7	2	5	1	6
Cereal and grain products	2	3	1	6	4	5	7
Convenience foods	6	2	3	5	7	1	4
Dairy	7	1	4	3	2	5	6
Edible oils and oil emulsions	5	2	1	4	3	6	7
Fruits, vegetables, nuts and legumes	3	1	7	5	2	4	6
Meat and meat products	5	2	7	4	3	1	6
Sauces, dressings, dips and spreads	5	2	3	7	6	1	4
Seafood and seafood products	1	5	6	7	3	4	2
Snack foods	2	1	3	6	7	4	5
Total sum	40	22	42	49	42	32	53
Average ranking	**3**	**1**	**4**	**6**	**4**	**2**	**7**

In the ranking, a greater number indicates higher sodium content; Hong Kong, the Hong Kong Special Administrative Region of the People's Republic of China.

### 3.3. Sodium content distributions

**Figure 2:**
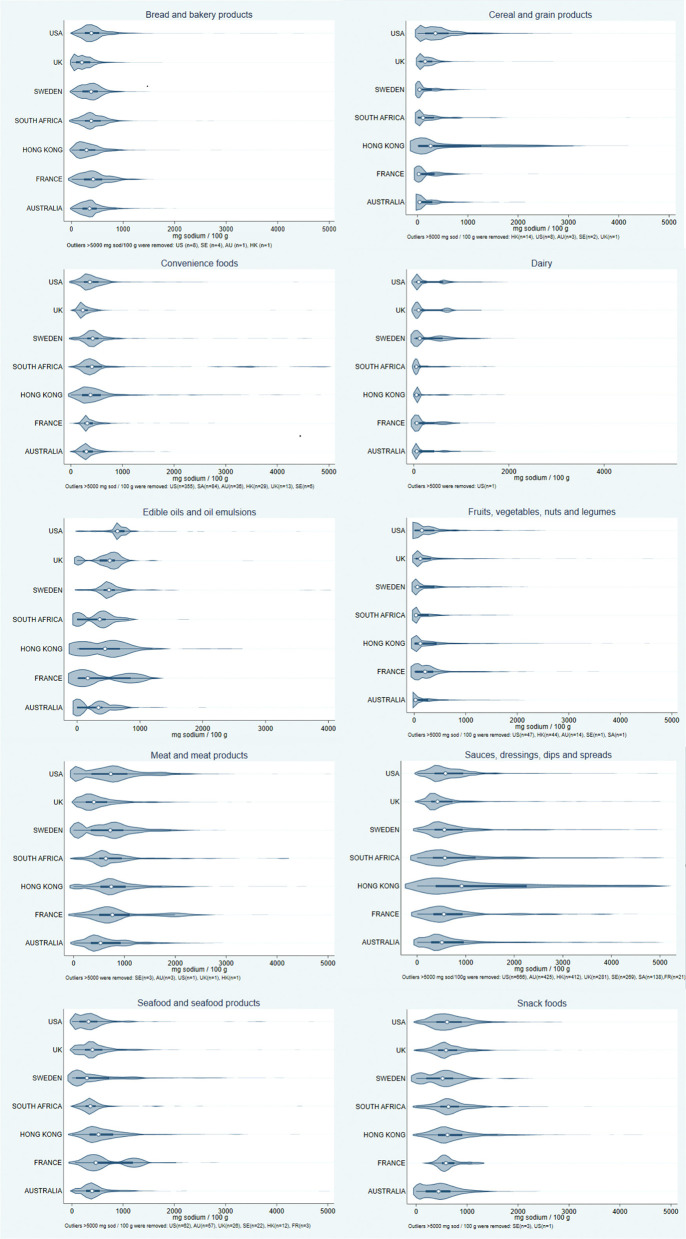
Violin plots showing the distribution of sodium content in food items by country in each food category. The categories containing most sodium were “meat and meat products,” “sauces, dressings, dips and spreads” and “snack foods.” Hong Kong, the Hong Kong Special Administrative Region of the People's Republic of China.

The Kruskal–Wallis test showed that there was a significant difference in sodium content between all countries in all food categories (*p* < 0.001). The distribution of sodium content in all food categories by country is displayed in Figure 2 and the following *post-hoc* pairwise comparison of means were significant.

In the “bread and bakery products” category, Sweden had higher sodium content than Australia, Hong Kong and United Kingdom (*p* < 0.0001), but lower than France, South Africa and United States (*p* < 0.002). Most Swedish food items in this category had a sodium content below ~900 mg per 100 g.

Swedish processed food items in the “cereal and grain products” category had lower sodium content than Australia, South Africa, the United Kingdom, Hong Kong and the United States (*p* < 0.0002). A majority of the Swedish food items had a sodium content under ~500 mg per 100 g.

Sweden had higher sodium content in the “convenience foods” category than all countries (*p* < 0.001) except South Africa (*p* < 0.006). Most Swedish food items in this category had a sodium content of < 600 mg per 100 g.

In the “dairy” category, Sweden had higher sodium content than Hong Kong, Australia, South Africa and France, but lower than the United States (*p* < 0.001). Most Swedish food items in this category had a sodium content below 200 mg per 100 g. In the Swedish data, the largest peak in the distribution was below the median, with a second peak by the third quartile, similar to the United States, the United Kingdom and France.

Sweden had higher sodium content than the United Kingdom (*p* < 0.02) in the “edible oils” and dairy products' category. Most Swedish items had sodium contents under 1,000 mg per 100 g. Unlike the other countries, the Swedish and United States distributions lacked a peak at the lower (left) end.

Swedish food items in the “fruits, vegetables, nuts and legumes” category had a higher sodium content than Australia and South Africa, but lower than France, Hong Kong, the United Kingdom and the United States (*p* < 0.005). Most Swedish food items in that category had a sodium content of <300 mg per 100 g.

In the “meat and meat products” category, Sweden had higher sodium content than the United Kingdom and Australia, but lower than all other countries (*p* < 0.0001). Most Swedish food items in this category had sodium contents approximately under 1,500 mg per 100 g, with the largest peak of the data distribution between the median and the third quartile. A second peak was found by ~100 mg sodium per 100 g, similar to the United States.

Sweden had a lower sodium content in the “sauces, dressings, dips and spreads” category than the United States and Hong Kong, but higher than France, Australia and the United Kingdom (*p* < 0.001). Most Swedish items had a sodium content below 1,200 mg per 100 g.

Sodium content in Swedish food items in the “seafood and seafood products” category was lower than South Africa, the United Kingdom, Australia, France and Hong Kong (*p* < 0.02). A majority of the Swedish food items in this category had a sodium content of <1,000 mg per 100 g.

Lastly, in the “snack foods” category, the sodium content in Sweden was higher than in Australia (*p* < 0.0005) and lower than in France, Hong Kong, South Africa, the United Kingdom, and the United States (*p* < 0.0002). A majority of the Swedish “snack foods” had a sodium content <1,000 mg per 100 g, with the largest peak of data distribution between the median and third quartile, and a second peak by ~50 mg per 100 g similar to Australia.

## 4. Discussion

### 4.1. Key findings

To the knowledge of the authors, this is the first study to examine the sodium content of packaged food items in Sweden, in several food categories compared to other countries. The three categories in Swedish data with the highest sodium content were “meat and meat products,” “sauces, dressings, dips and spreads” and “snack foods.” Based on median sodium content, food items in Sweden had more sodium than all or most of the included countries in the “dairy” and “convenience foods” categories. Food items in Sweden were on the other hand lower in sodium content than all or most of the included countries in the “cereal and cereal products,” “seafood and seafood products” and “snack foods” categories. Overall, Sweden was ranked in third place, i.e., having the third lowest sodium content overall of the included countries. Contrary to the initial hypothesis, processed food items in Sweden were not saltier than other countries' processed food items. This is however not equal to Swedish food items having a low sodium content.

### 4.2. Interpretation

In the Swedish data, some products in the categories with the highest sodium contents had values high enough to reach the recommended daily upper limit of sodium intake in just one portion. This was especially found in the portions of ready-made food, for instance, a portion of lasagna. Based on the median values of sodium content in food items in Sweden, it would separately take either 330 g of bacon or ham, 630 g of bread or 570 g of ready-made meals to reach the maximum recommended sodium intake of 2,400 mg. These results are consistent with information from the Swedish Food Agency. On their website, it is stated that two breakfast sandwiches with either ham or cheese contains 2.5 g salt (1,000 mg sodium; 42% of RDI), and that a ready-made meal of meatballs, potatoes and gravy contains 4.5 g salt (1,900 mg sodium; 80% of RDI) (31). This means that the recommended daily upper intake limit of sodium is reached before the last meal of the day in an average Swedish diet. That should be an indication that sodium content in Swedish food is too high from a health perspective. Since a diet consists of multiple different food categories, a lowering of sodium content is necessary in not just one, but several categories of food items.

Swedish data had the second highest sodium content of the seven analyzed countries in the “convenience foods” category. In this category, food products such as ready-made meals are found, a sub-category of convenience foods of which consumption has rapidly increased in Sweden in recent years (23). With Sweden already being one of the top consuming countries of ultra-processed foods in Europe (22), it is alarming that the sodium content in this food category was among the highest of the seven included countries. High consumers of ready-made meals have been shown to generally have lower compliance with nutrition recommendations (23). Lack of time affects food intake and eating (32, 33) and acts as a barrier for healthy diet behavior (31). An easy solution for lack of time to prepare meals from scratch is to consume ready-made meals or other types of convenience foods (33). A group that to a large extent consumes convenience foods is the elderly, especially those in single households (34). The reasons for this range from being too frail to cook, lack of perceived joy in cooking, or simply because it is easier to prepare or tastes better than food provided by home care services (35). Additionally, it has been shown that lunches that can be bought in Sweden from restaurants, grocery stores and fast-food chains contain a large amount of sodium (36). This means that the risk of consuming high sodium content from convenience is applicable on other groups in society, not exclusively the vulnerable. For example, Nordic studies show that the frequency of eating outside the home is positively associated with higher income (37). With that said, eating out in Sweden is far more uncommon than in Australia (38) and the United Kingdom (39).

Sweden would benefit from adapting the WHO SHAKE (Surveillence, Harness industry, Adopt standards for labeling and marketing, Knowledge, Environment) Technical Package for Salt reduction (40). SHAKE is designed to assist nations to reduce the sodium intake in the population, by step-by-step interventions targeting both industry, politics and consumers. The SHAKE package lifts the focus on from the individual consumer to the entire society, promoting a sodium reduction in all steps from food manufacturing to consumption.

The current sodium-lowering initiatives in Sweden do not fully help consumers that lack knowledge about the potential health consequences of a high salt intake. Neither do they help those that have no choice but to consume ready-made meals (39). It is an inadequate solution for governmental agencies to refer to front-of-pack labeling, or recommending buying less ultra-processed food, to lower population dietary sodium intake. This could only work if everyone has sufficient money, time, and skills to cook, and the health literacy to gauge the healthiness of food products.

The main identified problem based on the results of this study is that the sodium contents of processed food in Sweden are overall too high. As Swedes already eat twice as much sodium as the recommendations from the WHO and Swedish Food Agency (31, 41), there is a need for new policies targeting the producers of processed food items, as well as health literacy in the population (42).

The problem of a high sodium intake due to low socioeconomic status, often correlated with lower health literacy, is often stressed in low- and middle-income country settings. As previously discussed, high sodium intake and health literacy might also affect people with higher income. Therefore, this should also be taken into account in high-income settings, such as Sweden, when evaluating or introducing new policies. Based on the results of this study, the Swedish government should consider adopting other countries' initiatives, for example by setting sodium reduction targets that could significantly lower CVD burden (43), and providing more resources to increase consumer knowledge about the elevated risk of severe health consequences from an excessive sodium intake (44).

### 4.3. Strengths and limitations

This study's greatest strength is the magnitude of the databases and the wide geographical coverage of the included countries.

The FoodSwitch databases used in this study only contain packaged food items. Hence, any assumptions about dietary sodium intake, based on the results of this study, would only apply to people whose diet consists of mainly processed and packaged food items, those who obtain most of their meals outside of the home, e.g., at restaurants, and not those who prepare their meals solely from fresh food items. As the consumption of freshly prepared food is decreasing, the data in this study would therefore, to a large extent, be expected to reflect the dietary sodium content in the population (23). The published WHO benchmarks for sodium content in food (29) are not applicable on the food categories analyzed in this study, as the WHO benchmarks are narrower. Additionally, it is not possible to apply the recommended maximum intake of sodium on individual food items or food categories, as a diet generally consists of several different food categories. It is therefore hard to compare the sodium content in each food category from this study to the total recommended intake of sodium.

For the Swedish data, generalisability of the results should be considered high regarding sodium content in packaged and processed food items. Data were collected from four different retail databases, giving a width between different price classes, producers and target populations, even if there is a risk that packaged food items sold outside of these retailers have been missed. It is also not possible to determine intake of dietary sodium in the respective food category based on these results. For example, people might only eat the food items with sodium content lower, or higher, than the median values.

The representativeness of the other countries processed foods selection differs between countries. For Example, the United States dataset is updated regularly (with the data used in this study extracted in 2021) and considered to be representative of over 80 % of processed food items in the United States (45). That can be compared with the French dataset where data were collected from one grocery food retailer at one point in time. The external validity of the results of the present study for neighboring countries in the same region, or for other regions, is unknown. Global generalizability of the results would be increased if data from more countries were available to analyse. Future research should examine how products from only the top global manufacturers differ in nutritional content between regions, to see how much the same food items, from the same manufacturer, differ between countries.

A limitation is that the FoodSwitch database only carry information on food items in a limited number of countries currently reporting packaged product content to the FoodSwitch database.

Contrary to our hypothesis, processed food items in Sweden did not stand out as excessively high in sodium content in comparison to other countries. However, “dairy” and “convenience foods” had a high sodium content, and were ranked high in sodium compared to the other countries in our study. With the rapidly increasing in-take of salty processed food items, it is likely that consumers in Sweden will continue to struggle to stay below the national recommended maximum daily intake of sodium. Our results can assist policy interventions to reduce dietary sodium consumption.

## Data availability statement

The raw data supporting the conclusions of this article will be made available by the authors, without undue reservation.

## Author contributions

Conceptualization and investigation: KK, KR, and JS. Methodology, software, validation, and visualization: KK and JS. Formal analysis, writing—original draft preparation, and project administration: KK. Resources: BN and JS. Data curation: JS. Writing, review, and editing: KK, JS, KR, ED, JW, and BN. All authors have read and agreed to the published version of the manuscript.
