# Supplementary material for: Sodium content in processed food items in Sweden compared to other countries: a cross-sectional multinational study
Source: Front Public Health. 2023 Jun 8;11:1182132. doi: 10.3389/fpubh.2023.1182132 (PMC10287089; doi:10.3389/fpubh.2023.1182132)
Supplement: Supplementary file 1 [file Table_1.docx]

Supplementary Material

Sodium content in processed food items in Sweden compared to other countries: a cross-sectional multinational study

**Karin Karlsson1**†**, Karin Rådholm 2,3, Elizabeth Dunford 2,4, Jason HY Wu 2,5, Bruce Neal 2, *Johan Sundström 1,2**†

1 Department of Medical Sciences, Uppsala University, Uppsala, Sweden

2 The George Institute for Global Health, University of New South Wales, Sydney, Australia

3 Department of Health, Medicine and Caring Sciences, Linköping University, Linköping, Sweden

4 Department of Nutrition, Gillings Global School of Public Health, The University of North Carolina at Chapel Hill, USA

*** Correspondence:**Karin Karlsson

Karin.karlsson@uu.se

Johan Sundström
[johan.sundstrom@uu.se](mailto:johan.sundstrom@uu.se)

# Supplementary Table

General description of included food group categories

| **Food category** | **Sub-category** | **Description** |
| --- | --- | --- |
| **Bread and bakery products** | Bread | White, wholemeal and mixed grain/seed sliced bread and rolls Fruit bread and fruit-based muffins/rolls Wraps and other flatbread products Turkish pide, bagels, English-style muffins, crumpets, pizza bases and other plain bread-based products |
|  | Biscuits and cookies | Filled and unfilled sweet biscuits  Flavoured and plain crisp bread and crackers |
|  | Cakes, muffins and pastry | Scones, pikelets, doughnuts, cakes, sweet buns, pancakes, crepes, muffins, slices etc.  Cake, pikelet and pancake dry mixes Sweet pastries (fresh, ambient, chilled and frozen) |
| **Cereal and grain products** | Breakfast cereal | Ready to eat breakfast cereals Oats and other breakfast cereals that require heating  Other processed cereals (e.g. bran) |
|  | Cereal and nut-based bars | Plain, chocolate-topped and yoghurt-topped cereal-based bars |
|  | Noodles | Plain dry noodles Savoury/flavoured dry noodle-based dishes |
|  | Pasta | Canned and ambient pasta and sauce (with and without meat) products (excludes frozen ready meals)  Packaged fresh pasta with sauce Savoury/flavoured dry pasta-based side dishes Plain dry pasta |
|  | Rice | Plain rice Savoury rice-based side dishes |
|  | Other breakfast cereals | All breakfast products not included in ‘Breakfast cereal’ |
|  | Other cereal products | Flour and other unprocessed cereals (e.g. polenta, couscous, bread crumbs, yeast) |
| **Convenience foods** | Pizza | Frozen and refrigerated pre-packed pizzas |
|  | Soup | Canned, chilled and ambient soup products |
|  | Ready meals | Frozen, chilled and ambient pre-prepared meals |
|  | Meal kits | Kits with ingredients to put meals together |
|  | Pre-prepared salads and sandwiches | Chilled pre-prepared salads and sandwiches |
|  | Other convenience foods | Other pre-prepared foods not included in above categories |
| **Dairy** | Cheese | Feta, haloumi, parmesan and other high-salt cheeses  All types of full and reduced fat cheddar/Colby etc. cheese including shredded, block or sliced  Soft cheeses such as cream cheese, ricotta and cottage cheese  Processed cheese slices and products |
|  | Cream | Thickened, sour and regular cream products |
|  | Dairy desserts | Dairy-based desserts (e.g. custards, rice puddings)  Dairy-based dessert mixes (e.g. powders) |
|  | Ice cream and edible ices | Dairy and non- dairy-based ice cream varieties and edible ices |
|  | Milk products | Flavoured and unflavoured dairy milk products  Flavoured and unflavoured soymilks  Flavoured and unflavoured oat, almond and other milks  Condensed, evaporated and powdered milk products (including coconut milk) |
|  | Yoghurt products | Fruit, flavoured and natural yoghurts including yoghurt drinks |
| **Edible oils** |  | Salted and unsalted butter and margarine products |
| **Fruit, vegetables, nuts and legumes** | Fruit | Dried fruit products including coconut  Fruit-based bars  Fruit products canned in juice or syrup  Fruit gels, fruits in jelly and fruit puree |
|  | Jam and fruit spreads | Jams, marmalades and other preserves |
|  | Nuts and seeds | Salted and unsalted nuts and seeds |
|  | Vegetables | Canned tomato products  Canned beans and peas  Baked beans in tomato sauce  Canned creamed, plain and sweet corn  All other canned vegetables  Pickled vegetable and olive products  Frozen potato-based products  Frozen vegetables |
| **Meat and meat alternatives** | Meat alternatives | Plain tofu and other meat-free alternatives  Meat-free products (e.g. meat-free sausages) |
|  | Processed meat | Pre-packed bacon products  Beef, pork, chicken and lamb sausages and chilled hot dogs  Pre-packaged sliced deli meats  Pre-packaged salami and cured meats  Beef, pork, chicken and lamb meat burgers  Canned meat products (excluding soup and pasta)  Frozen meat pies, sausage rolls and other meat-based pastry products |
| **Sauces, dressings, dips and spreads** | Mayonnaise and salad dressings | Full and low-fat mayonnaise  Oil-based, vinegar-based and other types of salad dressing |
|  | Sauces | Table sauces such as tomato sauces and ketchups, sweet chili, BBQ sauces  Steak, HP and Worcestershire sauces  Soy, fish oyster and other Asian high-salt sauces  Mustard products  Marinade products  Meat accompaniments (e.g. apple, cranberry and mint sauces)  Plain and flavoured tomato paste products  Asian and Indian flavoured powdered, ambient and liquid meal-based sauces  Ambient and fresh pasta sauces  Recipe bases  Liquid and powdered gravies and stock |
|  | Spreads | Crunchy and smooth salted and unsalted peanut butter  Relishes, chutneys and pickles  Other savoury spreads (e.g. vegetable spreads)  Pâté spreads  Sweet spreads  Yeast-extract spreads (e.g. vegemite)  Chilled and ambient dips and salsa |
| **Seafood and seafood products** | Canned seafood | All varieties of plain and flavoured canned seafood |
|  | Chilled and frozen seafood | Chilled processed fish products (e.g. smoked salmon)  Coated frozen fish products (e.g. fish fingers) and uncoated fish products |
| **Snack foods** |  | Plain and flavoured potato crisps  Plain and flavoured snack foods  Extruded snacks (e.g. cheesy snacks)  Plain and flavoured corn chips  Pretzels, popcorn and other snack foods  Other fried snack foods (e.g. plantain chips)  All varieties of cracker-based snack packs |
